# Non-Invasive Tools to Detect Smoke Contamination in Grapevine Canopies, Berries and Wine: A Remote Sensing and Machine Learning Modeling Approach

**DOI:** 10.3390/s19153335

**Published:** 2019-07-30

**Authors:** Sigfredo Fuentes, Eden Jane Tongson, Roberta De Bei, Claudia Gonzalez Viejo, Renata Ristic, Stephen Tyerman, Kerry Wilkinson

**Affiliations:** 1 School of Agriculture and Food, Faculty of Veterinary and Agricultural Sciences, The University of Melbourne, Parkville, VIC 3010, Australia; 2 School of Agriculture, Food and Wine, The University of Adelaide, PMB 1, Glen Osmond, SA 5064, Australia

**Keywords:** bushfires, infrared thermography, near-infrared spectroscopy, smoke taint, artificial intelligence

## Abstract

Bushfires are becoming more frequent and intensive due to changing climate. Those that occur close to vineyards can cause smoke contamination of grapevines and grapes, which can affect wines, producing smoke-taint. At present, there are no available practical in-field tools available for detection of smoke contamination or taint in berries. This research proposes a non-invasive/in-field detection system for smoke contamination in grapevine canopies based on predictable changes in stomatal conductance patterns based on infrared thermal image analysis and machine learning modeling based on pattern recognition. A second model was also proposed to quantify levels of smoke-taint related compounds as targets in berries and wines using near-infrared spectroscopy (NIR) as inputs for machine learning fitting modeling. Results showed that the pattern recognition model to detect smoke contamination from canopies had 96% accuracy. The second model to predict smoke taint compounds in berries and wine fit the NIR data with a correlation coefficient (R) of 0.97 and with no indication of overfitting. These methods can offer grape growers quick, affordable, accurate, non-destructive in-field screening tools to assist in vineyard management practices to minimize smoke taint in wines with in-field applications using smartphones and unmanned aerial systems (UAS).

## 1. Introduction

A recent report from the Victorian government of Australia concluded that bushfires have increased in number and severity since the 1970s across the east and south of the country [1]. The main contributing factor to this environmental disaster is climate change, specifically the increased frequency of recurrent heat waves (i.e., prolonged periods of hotter weather) and drought conditions, which have increased the window of risk for bushfires, as well as their number, and severity. Recently, Chile (central region), USA (California), Greece, South Africa (Stellenbosch) and Australia (various states) have suffered some of the worst bushfires experienced in each country’s history. These countries are major producers of wines, and their grape growers and winemakers are similarly affected by global warming with detrimental effects in drought, vine phenological changes, shifting of suitable grapevine growing regions towards the north and south, and increased bush fire events near wine growing regions [2,3,4].

When bushfires occur in close proximity to vineyards, smoke can contaminate leaves and fruit. One of the main physiological effects of bush fire smoke in grapevines is the reduction of stomatal conductance (g_s_) [5]. Decreased g_s_ may be explained by the combination of the main smoke components carbon dioxide (CO_2_) and carbon monoxide (CO), with water vapor (100% RH in the substomatal cavity) producing carbonic acid (H_2_CO_3_) that reduces pH in the stomata, thereby causing partial or complete stomatal closure [5]. In berries, smoke contamination results in adsorption of smoke-derived volatile phenols (which accumulate in glycoconjugate forms), that are extracted into the final wine during the winemaking process [6]. Several mitigating measures have been evaluated to minimize smoke taint in berries or to remove volatile phenols (and their glycoconjugates) from wine, including defoliation [7] or foliar application of kaolin [8] in the vineyard, reverse osmosis treatment [9] and the addition of fining agents in wines [10]. However, implementation of defoliation or kaolin applications are often indiscriminate and broadly applied irrespective of the degree of grapevine exposure to smoke. Furthermore, the removal of smoke taint from wine is not selective and may inadvertently remove important wine compounds, thereby affecting the desirable organoleptic characteristics of wine.

Physiological assessment of control (non-smoked) and smoke affected grapevine cultivars have shown that some cultivars are more susceptible than others in terms of photosynthesis and stomatal conductance, in particular, Merlot and Cabernet Sauvignon. In contrast, Sauvignon Blanc was not significantly affected by smoke contamination from a physiological perspective. However, berries exposed to smoke resulted in wines with significantly higher concentrations of volatile phenols and guaiacol glycoconjugates compared to wines made from uncontaminated fruit [6].

The need to assess smoke contaminated fruit and wine has led to the implementation of new laboratory-based analytical methods [11], including liquid chromatography-tandem mass spectrometry for quantification of volatile phenol glycoconjugates [12,13]. However, these techniques require expensive laboratory instrumentation, and specialized technical skills to prepare samples (i.e., to extract the analytes of interest), operate the instruments and data analysis. Spectral methods, both mid-infrared (MIR) reflectance spectroscopy and chemometric techniques have been evaluated for rapid detection of smoke-taint in grapes [8] and bottled wines [14], but also with limitations. Spectral reflectance measurements of berries were affected by fruit maturity, while MRI-based classification of wines was influenced by cultivar, oak maturation and the level of smoke taint. Thus, reliable and rapid in-field techniques available to determine whether vines and fruits have been contaminated with smoke from bushfires are not yet available. This paper presents pattern recognition and regression models based on machine learning algorithms developed for the identification of smoke contaminated grapevine canopies and fruit. The first machine learning model generated used infrared thermography data from grapevine canopies as inputs to predict smoke contamination, as a target for four grapevine cultivars. Near infrared (NIR) spectroscopy readings from berries were used as inputs for regression machine learning algorithms to assess specific smoke-related compounds in berries and final wines from seven cultivars. These models combined with affordable geo-referenced NIR spectroscopy measurements of berries could allow growers to map contaminated areas of a vineyard to facilitate decision making at harvest. Finally, potential applications of these models using proximal and mid-range remote sensing using unmanned aerial systems (UAS) are discussed.

## 2. Materials and Methods

### 2.1. Experimental Site and Application of Smoke to Grapevines

**Figure 1:**
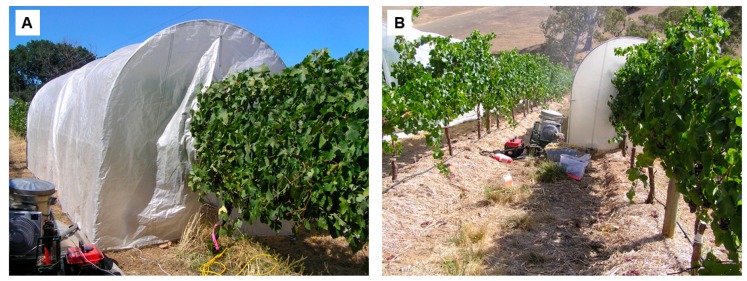
Grapevines were enclosed in a tent, and smoke from combustion of straw was blown into the tent using a fan. Pre-installation of the tent (**A**), and installed and operational tent (**B**). Photos obtained from the 2009/10 trial in Adelaide, Australia.

Grapevine smoke exposure experiments were conducted in the 2009/10 season using seven different cultivars grown at two locations: (i) Sauvignon Blanc, Pinot Gris, Chardonnay and Pinot Noir grown in a commercial vineyard in Adelaide Hills region, South Australia, Australia (35°00′ S, 138°49′ E) and (ii) Shiraz, Cabernet Sauvignon and Merlot vines grown in a vineyard located at the University of Adelaide’s Waite campus in Adelaide, South Australia (34°58′ S, 138°38′ E). Grapevines (three per replicate) were exposed to smoke (for 1 h) using a purpose-built smoke tent and experimental conditions described previously (Figure 1) [15]. Smoke was applied to vines at a phenological stage corresponding to approximately seven days post-veraison; when total soluble solids (TSS) concentrations were approximately 15 Brix, determined using a digital handheld refractometer (PAL-1, Atago, Tokyo, Japan).

Experiment 1 consisted of the physiological assessment of smoke contamination at the canopy level using porometry, infrared thermography, and pattern recognition machine learning using four cultivars two hours after smoke exposure: Chardonnay, Merlot, Sauvignon Blanc, and Shiraz. Experiment 2 assessed smoke taint in berries and wine at harvest for all seven cultivars. In this experiment, control (unsmoked) and smoke-affected berry samples (two berries taken from the mid-section of two bunches from two replicates per treatment, per cultivar; *n* = 112 berries) were collected at harvest. Morphometry of berries was measured using a caliper to obtain diameter (equatorial length in cm); length (cm) and calculated radius (cm), area (cm^2^), and perimeter (cm).

### 2.2. Experiment 1

#### 2.2.1. Physiological Measurements Using Leaf Porometry

Leaf conductance to water vapor was measured as stomatal conductance (g_s_) using a porometer (AP4, Delta-T Devices, Cambridge, UK). Porometer readings used were obtained from the cultivars Shiraz, Sauvignon Blanc, Chardonnay, and Merlot. Measurements were performed two hours after smoke treatments using nine mature, fully expanded sunlit leaves, for each of the two middle vines of two replicates per treatment per cultivar (*n* = 72) under natural leaf orientation with natural light intensity. Leaves were chosen to ensure measurements were performed on three leaves from the top, middle, and bottom parts of the canopies from each vine in a 3 × 3 matrix arrangement.

#### 2.2.2. Infrared Thermal Imagery of Canopies

**Figure 2:**
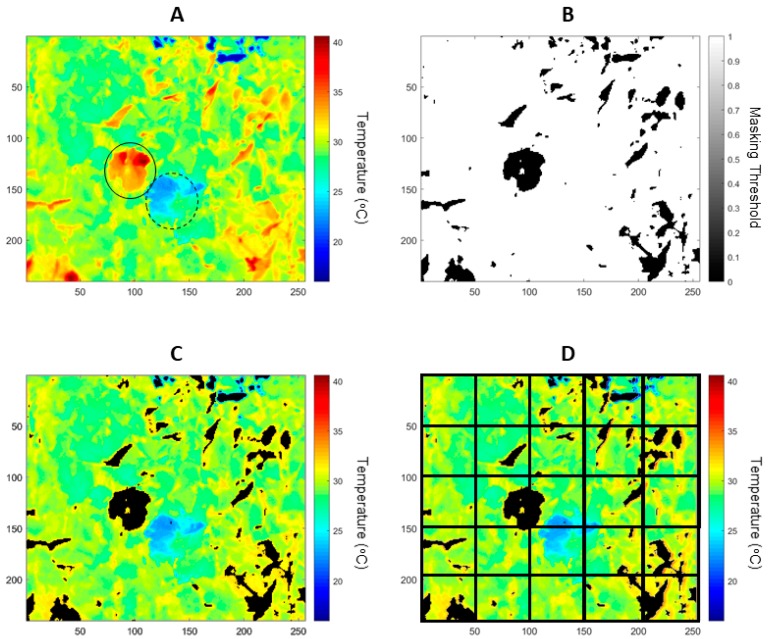
Examples of a radiometric thermal image (**A**) processing for data extraction of T_dry_ (A, solid circle) and T_wet_ (A, dotted circle) by painting leaves with petroleum jelly and water, respectively. Binary image obtained by thresholding T_dry_ and T_wet_ (**B**); masked radiometric image extracting non-leaf material, such as overheated elements and sky (**C**); and subdivision of thermal image to extract information from sections of the canopy in a 5 × 5 sub-division (**D**).

Thermal images were acquired from grapevine canopies using an infrared thermal camera FLIR^®^ T-series (Model B360) (FLIR Systems, Portland, OR, USA), with a resolution of 320 × 240 pixels. The camera measures temperature in the range of −20 to +1200 °C. The thermal sensitivity of the camera is <0.08 °C @ +30 °C/80 mK with a spatial resolution of 1.36 milliradians. Each pixel is considered an effective temperature reading in degrees Celsius (°C). Infrared thermal images were acquired from the same side and in parallel to porometer measurements (shaded side of the canopy to reduce variability) in the estimation of the infrared index (I_g_), which is proportional to g_s_ [16]. One thermal image from the canopy of each of the middle vines of two replicates per treatment per cultivar was obtained from a constant distance of 2.5 m perpendicular to the row direction (distance between rows being 3 m; Figure 2A). The calculated infrared thermal index (I_g_) was compared with porometry measurements acquired immediately after obtaining each thermal image from corresponding vines. All thermal images were acquired on a clear day. The smoke treatments were applied with minimal wind; a requirement for undertaking the field trials implemented to avoid the risk of fire spreading from accidental burning of interrow dry plant material and to secure representativeness of thermal images [16,17].

#### 2.2.3. Algorithms Used to Calculate Crop Water Stress Indices (CWSI) and Infrared Index (I_g_)

Crop water stress index (CWSI) was calculated using the following equation, after determining *T_dry_* and *T_wet_* [18]:

(1) $$\mathrm{CWSI}=\frac{T_{canopy}-T_{wet}}{T_{dry}-T_{wet}}$$

where *T_canopy_* is the actual canopy temperature extracted from the thermal image at determined positions, and *T_dry_* and *T_wet_* are the reference temperatures (in °C), obtained using the method of painting both sides of reference leaves with petroleum jelly and water, respectively [16].

An infrared index (*I_g_*), proportional to leaf conductance to water vapor transfer (*g_s_*), can be obtained using the relationship as follows [19]:

(2) $$I_{g}=\frac{T_{canopy}-T_{wet}}{T_{dry}-T_{wet}}=\;g_{s}\;(r_{aw}+\left(\frac{s}{ϒ}\right){\;}_{r_{HR}})$$

where *r_aw_* = boundary layer resistance to water vapor, *r_RH_* = the parallel resistance to heat and radiative transfer, ϒ = psychrometric constant and *s* = slope of the curve relating saturation vapor pressure to temperature [17,19].

#### 2.2.4. Infrared Thermography Data Extraction

The T_dry_ and T_wet_ values were obtained on a per image basis using a customized code written in Matlab^®^ R2019a (Mathworks Inc. Natick, MA, USA) to crop the radiometric data from the areas within the respective painted leaves with water (T_wet_) and petroleum jelly (T_dry_) (Figure 2A). To filter non-leaf material from the radiometric image using the determined threshold, a second customized code was written in Matlab® to binarize a masked image (Figure 2B) and to extract these values from the original image (Figure 2C). For automatic extraction of data within a canopy, a pre-defined subdivision of 3 × 3 = 9; 5 × 5 = 25; 7 × 7 = 49 and 10 × 10 = 100 was automatically implemented (Figure 2D; for the case of 5 × 5). From these subdivisions, data were extracted for T_canopy_ per image (Figure 2D), I_g_ Equation (2) and CWSI Equation (1).

The image sub-divisions (Figure 2D) represent the matrix (A) with *m* × *n* (*m* = rows and *n* = columns) extraction points represented as per the following matrix:

(3) $$A\left(m,n\right)=\;\left(\begin{array}{ccc} T_{1,1} & \cdots & T_{1,n}\\ \vdots & \ddots & \vdots \\ T_{m,1} & \cdots & T_{m,n} \end{array}\right)$$

Since *m*, *n* represent the pre-determined subdivision for automatic cropping sections from the infrared thermal image (A), every sub-image is processed for automatic canopy extraction by filtering non-leaf temperatures using the T_dry_ and T_wet_ values extracted (Figure 2B) as minimum and maximum possible temperatures for the canopy. The calculated T value then corresponds to the averaged T_canopy_ for each sub-division.

#### 2.2.5. Pattern Recognition of Infrared Thermal Imagery using Machine Learning for Smoke Contamination Prediction

**Figure 3:**
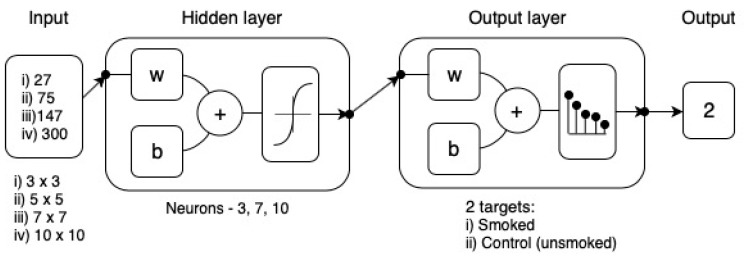
Diagram of the two-layer feedforward network with a tan-sigmoid function in the hidden layer and a Softmax transfer function in the output layer. For hidden and output layers, *w* = weights and *b* = biases. Input volume and neuron trimming exercises are included in the diagram.

Pattern recognition models were developed using a customized Matlab® code, which is able to test 17 different training algorithms, two from Backpropagation with Jacobian derivatives, 11 from Backpropagation with gradient derivatives and four from Supervised weight and bias training functions, in loop to select the best model. This model was constructed using the infrared thermal image output values as inputs to classify the samples into smoked and non-smoked (control). The infrared thermal images were analyzed with the methodology described in Figure 2 to obtain T_canopy_, I_g_, and CWSI data obtained using Equations (1) and (2) with sub-divisions of 3 × 3 (*n* = 27 per image); 5 × 5 (*n* = 75, per image; 7 × 7 (*n* = 147, per image) and 10 × 10 (*n* = 300 per image). All algorithms tested used a random data division. However, for the algorithms such as scaled conjugate gradient, which consist of three stages—training, validation and testing, the data was divided as 60% (*n* = 28 images) for training, 20% (*n* = 10 images) for validation with a cross-entropy performance algorithm, and 20% (*n* = 10 images) for testing with a default derivative function. For the algorithms such as sequential order weights and bias, which only consist of training and testing stages, the data was divided as 70% (*n* = 34) for training and 30% (*n* = 14) for testing with a cross-entropy performance algorithm. A trimming exercise was conducted using 3, 7 and 10 neurons to select the best model with no signs of overfitting (Figure 3).

### 2.3. Experiment 2

#### 2.3.1. Berry Near Infrared (NIR) Spectroscopy Measurements

Full berries were scanned using a spectrophotometer (ASD FieldSpec^®^3, Analytical Spectral Devices, Boulder, CO, USA) equipped with the ASD contact probe, built for contact measurements, attached by fiber optic cable to the instrument. A total of 112 berries collected at harvest from seven cultivars (16 berries per cultivar) were scanned by putting the probe’s lens in contact with the berries and a total of 401 spectra were recorded for each berry. The instrument records spectra with a resolution of 1.4 nm for the region 350–1000 nm and 2 nm for the region 1000–1850 nm. The instrument was used in reflectance mode and data was then transformed into absorbance values (absorbance = log (1/reflectance)). A reference tile (Spectralon^®^, Analytical Spectral Devices, Boulder, CO, USA) was used as a white reference, for scatter correction. A new reference was taken every ten spectra acquisitions.

#### 2.3.2. Winemaking and Chemical Analysis of Berries and Wine

Small scale winemaking of control and smoke-affected fruit from this trial has been described previously in detail by Ristic et al. [6]. Guaiacol glycoconjugates were measured in fruit and wine by HPLC–MS/MS using a stable isotope dilution analysis (SIDA) method developed by Dungey et al. (2011) [12]. Volatile phenols, including guaiacol, were determined in berries and wine by the Australian Wine Research Institute’s (AWRI) Commercial Services Laboratory (Adelaide, Australia). Volatile phenols were measured by GC–MS according to SIDA methods reported previously [13].

#### 2.3.3. Fitting Modeling of Near-Infrared (NIR) Spectroscopy of Berries Using Machine Learning Modeling to Predict Smoke Taint in Berries and Wine

**Figure 4:**
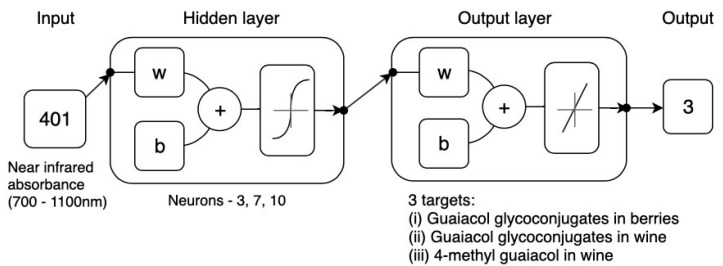
Diagram of the two-layer feedforward network with a tan-sigmoid function in the hidden layer and a linear transfer function in the output layer. For hidden and output layers, *w* = weights and *b* = biases. Neuron trimming exercise is included in the diagram.

A regression model was developed using a customized Matlab^®^ code, which is able to test 17 different training algorithms, two from Backpropagation with Jacobian derivatives, 11 from Backpropagation with gradient derivatives and four from Supervised weight and bias training functions, in loop to select the best model. NIR absorbance values corresponding to the range of wavelengths within 700 and 1100 nm with a second derivative transformation, which were used as inputs in the machine learning algorithms, since that range corresponds to alcohol and alcohol-based compounds to predict (i) guaiacol glycoconjugates in berries (µg Kg^−1^), (ii) guaiacol glycoconjugates in wines (µg L^−1^) and iii) guaiacol in wine (µg L^−1^). All algorithms tested used a random data division. However, for the algorithms, which consist of three stages—training, validation and testing, the data was divided as 60% (*n* = 28) for training, 20% (*n* = 10) for validation with a means squared error (MSE) performance algorithm and 20% (*n* = 10) for testing with a default derivative function (data not shown). For the algorithms such as sequential order weights and bias, which only consist of training and testing stages, data was divided as 70% (*n* = 34) for training and 30% (*n* = 14) for testing with a means squared error performance algorithm. A trimming exercise was conducted using 3, 7, and 10 neurons to select the best model with no signs of overfitting (Figure 4).

### 2.4. Statistical Analysis

Data from chemometry and morphometry of berries, wine compounds, and I_g_ and g_s_ were analyzed through ANOVA using SAS^®^ 9.4 software (SAS Institute Inc., Cary, NC, USA) with Tukey’s studentized range test (HSD; *p* < 0.05) as post-hoc analysis for multiple comparisons to assess significant differences. Statistical data such as means and standard deviation (SD) were obtained from the replicates of each cultivar and treatment.

## 3. Results

### 3.1. Experiment 1

#### 3.1.1. Grapevine Physiological Data Relationships between Porometry and Infrared Thermal Imagery

Table 1 shows the mean values for g_s_ and I_g_ with respective standard deviations (SD) for the four cultivars from Experiment 1. The general trend for the mean values of the control treatments follows a positive linear relationship (R^2^ = 0.99; I_g_ = 0.0027 g_s_). On the contrary, the trend for the mean values of the smoke treatments have lower linearity and relationship, but still showed a positive linear pattern (R^2^ = 0.23; I_g_ = 0.0023 g_s_; data not shown). In the control samples, the mean Ig values per cultivar did not show significant change, as reflected by the SD values, but Merlot showed a significantly higher I_g_ (*p* < 0.05). This trend was similar for the mean g_s_ values showing Merlot with the highest mean (*p* < 0.05). The mean I_g_ values for smoked samples were more variable, while g_s_ showed higher mean values, except Sauvignon Blanc, and more variability as reflected by the higher SD values compared to control. The I_g_ mean values for both treatments were not very sensitive, as seen in Table 1.

**Figure 5:**
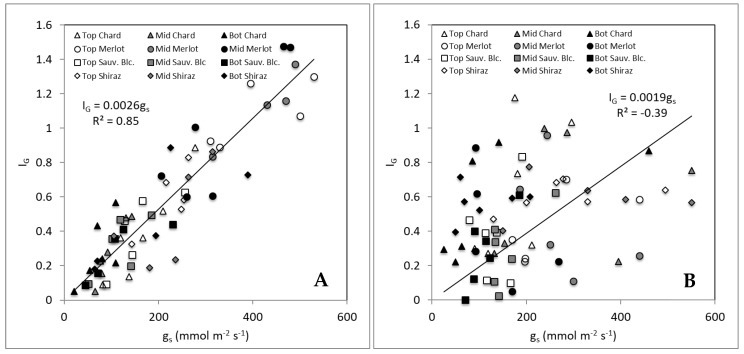
Relationship between g_s_ and I_g_ for Experiment 1 in the four cultivars with data separated between canopy sections: top (Top), middle (Mid) and bottom (Bot) measurements for control treatments (**A**) and smoked treatments (**B**).

Figure 5 shows the relationships between g_s_ and I_g_ for different sections of canopies (top, middle, and bottom) of the grapevines monitored for both non-smoked (control) and smoked treatments. The graph (Figure 5A) shows a strong and significant linear relationship between g_s_ and I_g_ (R^2^ = 0.85; I_g_ = 0.0026 g_s_). However, there was no relationship observed for smoke treatments, with the data presenting high variability, which is consistent with results shown in Table 1. Figure 5A shows that regardless of the measurement position within the canopy for I_g_, there is a broader distribution of values between top, middle, and bottom of the canopy along the linear relationship found. On the contrary, Figure 5B shows that the bottom readings for g_s_ are more concentrated towards the lower values (<200 mmol m^2^ s^−1^). Furthermore, the Ig values become less sensitive (spread between 0 and 1). The same pattern can be seen for most of the top readings with the middle readings having a wider spread distribution.

#### 3.1.2. Pattern Recognition Using Machine Learning Modeling of Physiological and Infrared Thermal Data

Table 2 shows the results of the pattern recognition modeling for the data extracted from infrared thermal images from the canopies of four different cultivars combined for Experiment 1. The best performing algorithm for the 3 × 3 sub-division and extraction of T_canopy_, I_g_, and CWSI used as inputs and classification of smoked and non-smoked as target was the scaled conjugate gradient algorithm. The training, validation, and testing procedures (using 10 neurons) resulted in an overall model with 94% accuracy. In the case of the data extracted using a 5 × 5 sub-division, the overall best model (sequential order weight and bias) resulted in an accuracy of 88% (using 10 neurons) in the classification of smoked and non-smoked canopies. For the 7 × 7 sub-division, the best algorithm (also the sequential order weight and bias) resulted in an accuracy of 94% (using 7 neurons) in the classification. Finally, the 10 × 10 was the best performing algorithm overall (sequential order weight and bias) resulted in an accuracy of 96% (using 3 neurons). Furthermore, the performance of training was lower than the one for testing, and testing accuracy was close to that from the training stage, which are evidence of no overfitting [20,21].

**Figure 6:**
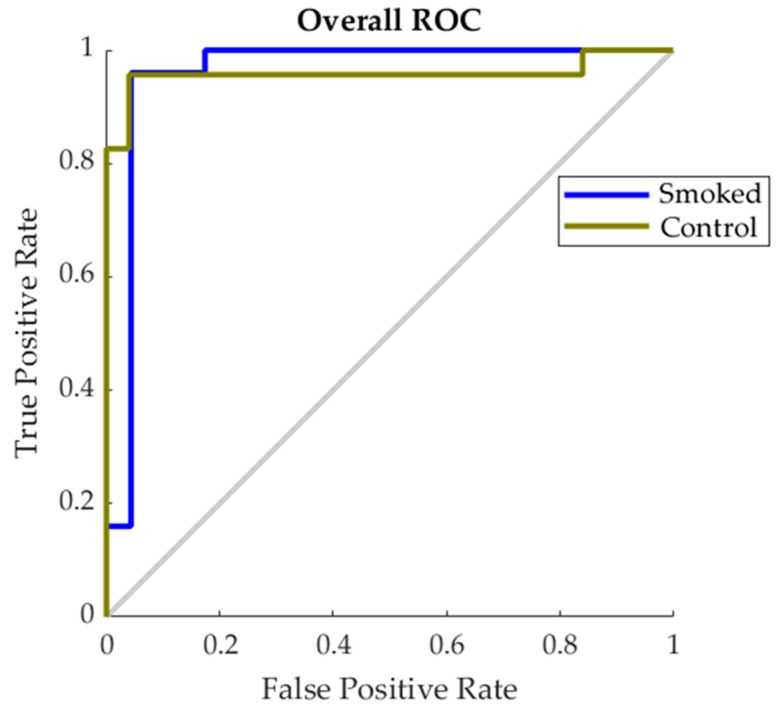
Receiver Operating Characteristic (ROC) showing the false positive rate (*x*-axis) and true positive (*y*-axis) for control and smoked treatments for the best performing classification model found in Table 3.

Figure 6 shows the Receiver Operating Characteristic (ROC) for the best performing model found to predict smoke contamination in grapevine canopies (10 × 10 sub-division; Table 2). The figure shows that results for both smoke and control pattern recognition using infrared thermography data as inputs are projected in a similar trend to the True Positive Rate prediction axis of the graph.

### 3.2. Experiment 2

#### 3.2.1. Berry Morphology and NIR Peak within the 700–1100 nm

Table 3 shows the average data of morphometric and chemometric measurements obtained from berry samples for all the seven cultivars for Experiment 2. Even though there are some significant differences between morphometric measurements of berries for the different cultivars comparing smoke and non-smoked (Control) treatments, they do not affect results and models developed.

#### 3.2.2. Smoke-Related Compounds Found in Berries and Wines

Data for smoke-related compounds have been previously reported by Ristic et al. (2016) [6], and comprised of volatiles with statistical differences between control (non-smoked) and smoked treatments. Specifically, for purposes of modeling, guaiacol glycoconjugates found in berries (µg Kg^−1^), guaiacol glycoconjugates found in wines (µg L^−1^) and guaiacol found in wines (µg L^−1^) were used since these are the primary compounds identified by the industry to contribute to smoke taint. In berries, the guaiacol glycoconjugates average concentration ranged for control between 37 and 602 µg kg^−1^ and from 253 to 2452 µg kg^−1^ for smoke-affected treatments. The guaiacol glycoconjugates concentrations in wines ranged from 8 to 334 µg L^−1^ for control and from 111 to 1480 µg L^−1^ for smoke-affected treatments. In the case of guaiacol concentration in wines, values ranged from 0 (not detected) to 9 µg L^−1^ for control and from 0 (not detected) to 26 µg L^−1^ [6].

#### 3.2.3. Near-Infrared (NIR) Spectroscopy from Berries and Smoke Taint Compounds Found

**Figure 7:**
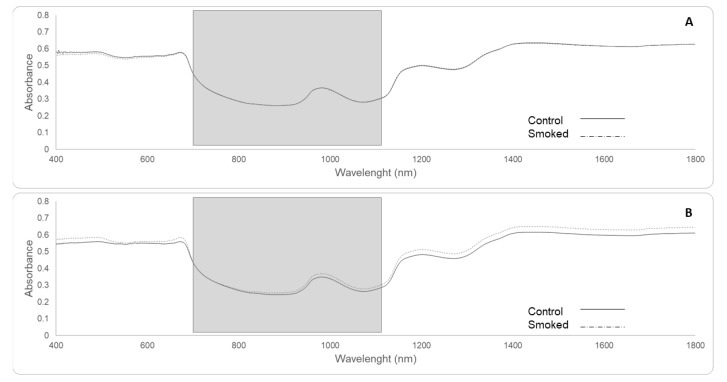
Average spectra for control (solid line) and smoke-affected berries (dashed line) from red cultivars (**A**): Merlot, Shiraz, Pinot Noir, Cabernet Sauvignon and white cultivars (**B**): Pinot Gris, Chardonnay and Sauvignon Blanc. The grey rectangles represent the wavelengths used for machine learning fitting modeling (700–1100 nm), with the main peak at 982 nm.

Figure 7 shows the main average spectra for berries from smoke and non-smoked (control) treatments for red (Figure 7A) and white cultivars (Figure 7B). There were no significant differences in the averaged spectra between smoked and non-smoked berries for red cultivars. On the contrary, there appears to be a consistent difference for white cultivars of around 0.05 in absorbance, especially from 820 to 1100 nm for the range considered for this study. Smoke-related compounds for this trial and used for the machine learning model reported here have been previously reported by Ristic et al. [6]. In this study, statistically significant differences in the main smoke taint compounds were reported for all the seven cultivars included in Experiment 2.

#### 3.2.4. Machine Learning Modeling Based on NIR Spectra to Estimate Smoke Taint Compounds in Berries and Wine

**Figure 8:**
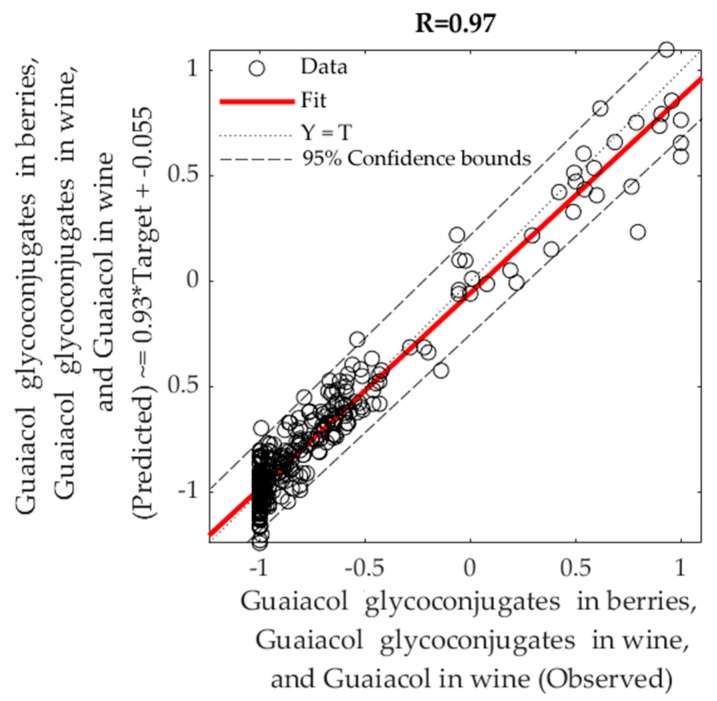
Overall fitting model using machine learning (Sequential Order Weights and Bias) using NIR spectra (700–1100 nm; second derivative transformation) of berries from seven grapevine cultivars as inputs and main smoke taint compounds found in berries and wine as targets.

Table 4 shows the best machine learning regression model obtained for the NIR data from berries (700–1100 nm using the second derivative transformation; Sequential Order Weights and Bias) as inputs and smoke taint compounds measured in berries and wine. The correlation between the estimated and observed values was R = 0.97 and slope b = 0.93 (close to unity). The same correlations and similar slopes were found for the training and the test stages. The overall model can also be seen in Figure 8, in which most of the point cloud data fits in the 1:1 line representing the accuracy of predicted versus observed data. Based on the 95% confidence bounds, the overall model had 3.6% of outliers. The performance of training was lower than the one for testing, and testing accuracy was the same as that from the training stage, which are evidence of no overfitting [20,21].

## 4. Discussion

### 4.1. Physiological Changes within Grapevine Canopies Due to Smoke Contamination

The relationship between the I_g_ thermal index and g_s_ is linear, as shown in Table 1 and Figure 5A for non-smoked vines. These results are consistent with other studies showing the same relationships for grapevines [16,17], coffee plants [22] and olive trees [23], which are tree-like or bushy canopies. However, this relationship was not observed for smoked canopies of the four cultivars from Experiment 1 (Figure 5B). Smoke contamination is an external signal to the plant which is composed mainly of CO, CO_2_ and other gases, which cause acidification of the sub-stomatal cavity due to the production of carbonic acid (H_2_CO_3_) when combined with water, with the resulting pH reduction causing partial or complete stomata closure [5]. This effect could explain the increased variability within g_s_ data amongst individual leaves that was detected in porometry data (Table 1 and Figure 5B). The reported I_g_ data from the whole infrared thermal images (Table 1) did not have significant differences in the variability of the data, which can be explained by the unrepresentativeness of means when using this type of high-resolution information.

It is important to note that the comparison between g_s_ and I_g_ for Figure 5 was made in this case using the methodology proposed in Figure 2 and with a sub-division of 3 × 3 for comparison purposes. Since every image was taken from 2.5 m distance, the field of view from infrared thermal images was around 140 × 110 cm of the canopy, which divided by nine gives a sub-area of 47 × 37 cm (area = 1739 cm^2^). Considering that the area of an average leaf (data not shown) is of around 50–80 cm^2^ [24], the I_g_ values represent the average of an area of approximately 25-fold of single leaves, in which porometry was conducted. This difference may explain the lower sensitivity of I_g_ to g_s_, especially for smoked canopies with higher g_s_ variability expected even at the leaf level (patchy stomata behavior).

The extraction of I_g_ values from infrared thermal images require a T_dry_ and T_wet_ reference temperatures. In this study, the painted leaves method was implemented for more accuracy in the determination of reference temperature thresholds to separate leaf from non-leaf material in the analysis. However, this method is manual and hinders the possibility of automation. Alternatively, the leaf energy balance method could be implemented using micrometeorological weather data such as temperature, relative humidity, and solar radiation to calculate T_dry_ and T_wet_ on-the-go, while obtaining the infrared thermal images. It is common nowadays to access cheap sensor technology to measure these micrometeorological variables and dataloggers or access to the Internet of Things (IoT) for data transmission and processing. Previous research has shown that these reference temperatures can be calculated with high accuracy (R^2^ = 0.95; RMSE = 0.85; *p* < 0.001) [16]. Furthermore, there is the requirement for infrared thermal images to be explored and assessed more in-depth at higher subdivisions and using machine learning modeling to assess the pattern variability and use it as a predictor of smoke contamination levels.

### 4.2. Pattern Recognition of Smoke Contamination Using Machine Learning Modeling

Considering the sub-division of infrared thermography data, the field of view of canopies and size of single leaves for this study, it is not surprising that the best pattern recognition model (96% accuracy) using machine learning (Sequential order weight and bias) was obtained with the 10 × 10 subdivision. This sub-division will render comparison areas within the canopy of 154 cm^2^, which is only 2.2-fold compared to a single leaf area (70 cm^2^). Furthermore, from the neuron trimming analysis, a highly accurate model was obtained for the classification of smoked and non-smoked canopies with three neurons, which makes the model more efficient and less susceptible to overfitting. The latter is also supported by the performance value obtained by this model. Results shown in this paper from pattern recognition modeling using machine learning to asses smoke contamination of canopies have excellent potential for the use in short and mid-range remote sensing based on Unmanned Aerial Vehicles (UAVs) platforms. From Figure 5B, it can be seen that the main variability within g_s_ values is in the bottom and top parts of the canopies, which validates obtaining infrared thermal imagery using UAVs at 0° Nadir angle. Furthermore, models developed in this study should be tested using UAV with infrared cameras that could render a 15 × 15-pixel resolution, which corresponds to an area of 225 cm^2^, which is close to the 154 cm^2^ area used for machine learning modeling here.

**Figure 9:**
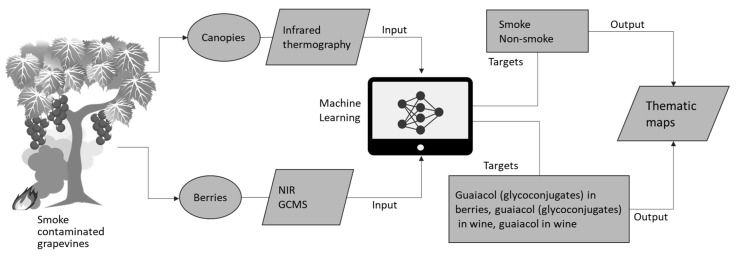
Diagram showing the implementation of machine learning modeling strategies proposed in this paper for proximal (using smartphones and portable infrared thermal cameras and NIR devices) and mid-distance remote sensing using unmanned aerial system (UAS) platforms.

This kind of remote sensing tool can render spatial distribution maps of contaminated areas within vineyards that could aid growers to apply differential management strategies discussed before to mitigate smoke contamination of the fruit. Spatial maps of smoke contamination can also help to achieve differential harvests to avoid mixing fruit with smoke-tainted fruit. Hence, a system is proposed using these methods, which is depicted in Figure 9 for proximal and mid-distance remote sensing using infrared cameras and UAV platforms. For proximal remote sensing, the algorithms developed in this study can be implemented in smartphone devices as computer applications (Apps) connected to portable and affordable infrared thermal cameras (i.e., FLIR One^®^, FLIR Systems, Portland, OR, USA) and near-infrared spectroscopy devices (i.e., Lighting Passport^®^, AsenseTek, Taipei, Taiwan).

### 4.3. Near-Infrared (NIR) Spectroscopy of Berries

Since NIR spectroscopy was obtained from full berries, the tool proposed in this paper is non-destructive. Furthermore, it has been shown that a higher concentration of smoke-related compounds after contamination can be found in the skin of berries, which is higher than in the pulp and higher than the seeds [12]. Furthermore, the range of 700–1100 nm was chosen since most of the available NIR instrumentation in this range can be affordable for growers compared to the instrument used in this study which can cost around 45 times more. The 982 nm overtone is associated with the OH overtone band and 1100 for the CH bands, which corresponds to alcohol and phenolic compounds [25].

The model reported using machine learning fitting algorithms can be of great assistance to growers and winemakers to obtain chemometry data in real time using the proposed methodology shown in Figure 9. Currently, growers do not have sophisticated tools to assess potential smoke contamination of berries bunches and wines. The only option available is collection of samples within a vineyard for compositional analysis by an accredited laboratory using GC-MS or HPLC-MS/MS. This process is destructive, expensive, and takes a long time, which makes it less ideal for the implementation of mitigation strategies and/or decision making before harvest. Furthermore, it may minimize smoke taint by the information provided through a spatial assessment of the contamination either through canopies or berries for informed decision making regarding palliative measures (as presented in this paper) or differential harvest.

The models developed in this study were able to predict smoke contamination in canopies, berries and wines, regardless of the cultivar. Hence, the models could be applied as a universal methodology. Further studies and data acquired could be added to models to include more cultivars. However, the seven cultivars included in this study were some of the most commercially important in Australia. Finally, it is important to note that the levels of smoke-taint compounds present in wine are in part related to the winemaking process (i.e., duration of skin contact time during fermentation), hence this model will need to be adjusted for different winemaking techniques, which can influence the extraction of smoke-related compounds from the berry.

## 5. Conclusions

This paper showed two main advancements for tools to detect smoke contamination in grapevine canopies and smoke-related compounds in berries and wine using remote sensing techniques. This study is the first to apply machine learning modeling techniques to assist growers confronted with vineyard exposure to smoke from bushfires, an issue which has been exacerbated in prominent wine regions around the world due to climate change. Furthermore, this paper has proposed an affordable method to implement these novel techniques using smartphones, portable thermal imagery and NIR spectroscopy devices. More research is required to assess the usage of these affordable devices in the future using the models proposed.

## Figures and Tables

**Table 1:** Means and standard deviation (SD) per variety and treatment for the infrared index (Ig, unitless) and stomatal conductance (g_s_ in mmol m^2^ s^−1^) calculated for all the images without sub-divisions.

Variety	I_g_		g_s_ (mmol m^2^ s^−1^)		I_g_		g_s_ (mmol m^2^ s^−1^)	
	Control				Smoked			
	**Mean**	**SD**	**Mean**	**SD**	**Mean**	**SD**	**Mean**	**SD**
**Chardonnay**	0.32 ^b^	0.22	112.66 ^c^	60.55	0.60 ^a^	0.34	203.02 ^ab^	145.72
**Merlot**	1.06 ^a^	0.29	384.93 ^a^	102.68	0.43 ^ab^	0.28	251.00 ^a^	131.44
**Sauvignon Blanc**	0.34 ^b^	0.18	130.60 ^c^	60.12	0.32 ^b^	0.23	135.89 ^b^	46.49
**Shiraz**	0.52 ^b^	0.26	211.40 ^b^	88.97	0.59 ^a^	0.10	235.35 ^ab^	148.71

Means followed by different superscript letters are statistically significant between treatments based on Tukey’s studentized range test (HSD, *p* < 0.05).

**Table 2:** Best pattern recognition model developed for each set of inputs showing the best training algorithm and number of neurons to predict whether canopies are smoked or non-smoked (control). Inputs corresponds to data extracted from infrared thermal images for T_canopy_, I_g_ and crop water stress index (CWSI) in matrix arrangement of 3 × 3 (*n* = 27), 5 × 5 (*n* = 75), 7 × 7 (*n* = 147) and 10 × 10 (*n* = 300) data points per thermography. Performance reported is based on cross-entropy.

Inputs	Algorithm	Neurons	Stage	Samples	Accuracy	Performance
3 × 3	Scaled conjugate gradient	10	Training	28	100%	0.03
			Validation	10	90%	0.16
			Test	10	80%	0.44
			Overall	48	94%	-
5 × 5	Sequential order weight and bias	10	Training	34	85%	0.37
			Test	14	93%	0.43
			Overall	48	88%	-
7 × 7	Sequential order weight and bias	7	Training	34	94%	0.72
			Test	14	93%	0.71
			Overall	48	94%	-
**10 × 10**	**Sequential order weight and bias**	**3**	**Training**	**34**	**97%**	**0.45**
			**Test**	**14**	**93%**	**0.47**
			**Overall**	**48**	**96%**	**-**

**Table 3:** Morphometric data obtain from berries for all seven cultivars consisting in Perimeter (P in cm), Equatorial Diameter (D in cm), calculated Area (A in cm2) and Radius (D/2 in cm). For chemometry, Total Soluble Solids (TSS) represented by Brix and Near Infrared (NIR) absorbance at 982 nm corresponding to H–O–H and O–H chemical bonds.

	P (cm)		D (cm)		Area (cm^2^)		R (cm)		Brix (°)		NIR982 (nm)	
	C	S	C	S	C	S	C	S	C	S	C	S
**Merlot**	4.7 ^a^	4.6 ^a^	1.4 ^a^	1.3 ^a^	1.5 ^a^	1.5 ^a^	0.3 ^c^	0.3 ^bc^	23.9 ^a^	24.2 ^ab^	0.4 ^abc^	0.3 ^ab^
**Shiraz**	4.1 ^b^	3.9 ^bc^	1.3 ^ab^	1.2 ^b^	1.5 ^a^	1.2 ^c^	0.4 ^ab^	0.3 ^cd^	24.9 ^a^	25.4 ^a^	0.3 ^cd^	0.4 ^a^
**PinGr**	4.0 ^bc^	4.2 ^b^	1.3 ^bc^	1.3 ^ab^	1.4 ^ab^	1.5 ^a^	0.3 ^bc^	0.4 ^a^	18.7 ^c^	19.8 ^d^	0.3 ^abc^	0.4 ^a^
**Char**	4.0 ^bcd^	3.8 ^cd^	1.4 ^a^	1.3 ^ab^	1.5 ^a^	1.3 ^bc^	0.4 ^a^	0.3 ^abc^	19.7 ^cd^	18.6 ^de^	0.5 ^a^	0.4 ^a^
**PinNoir**	3.8 ^cd^	3.8 ^cd^	1.2 ^c^	1.3 ^ab^	1.2 ^bc^	1.3 ^abc^	0.3 ^c^	0.3 ^ab^	17.2 ^d^	18.2 ^e^	0.3 ^bcd^	0.4 ^a^
**CabSauv**	3.7 ^cd^	3.6 ^d^	1.1 ^d^	1.1 ^c^	1.1 ^c^	1.0 ^d^	0.3 ^d^	0.3 ^d^	24.1 ^a^	23.1 ^b^	0.4 ^ab^	0.4 ^a^
**SauvBl**	3.7 ^d^	3.8 ^c^	1.3 ^bc^	1.3 ^ab^	1.3 ^b^	1.4 ^ab^	0.4 ^ab^	0.4 ^a^	20.8 ^b^	21.5 ^c^	0.2 ^d^	0.2 ^b^

Abbreviations: C = Control, S = Smoke, PinGr = Pinot Gris, PinNoir = Pinot Noir, Char = Chardonnay, CabSauv = Cabernet Sauvignon, SauvBl = Sauvignon Blanc. Different superscript letters are statistically significant between treatments based on Tukey’s studentized range test (HSD, *p* < 0.05).

**Table 4:** Regression model using machine learning (Sequential Order Weights and Bias) for NIR data from berries of seven grapevine cultivars showing the correlation coefficient (R) and performance based on mean squared error (MSE) for each stage.

Stage	Samples	Observations	R	Slope	Performance (MSE)
**Training**	78	234	0.97	0.91	0.86
**Test**	33	99	0.97	0.96	0.91
**Overall**	111	333	0.97	0.93	-
